# Metabolic Reprogramming by Reduced Calorie Intake or Pharmacological Caloric Restriction Mimetics for Improved Cancer Immunotherapy

**DOI:** 10.3390/cancers13061260

**Published:** 2021-03-12

**Authors:** Erwan Eriau, Juliette Paillet, Guido Kroemer, Jonathan G. Pol

**Affiliations:** 1 Centre de Cancérologie de Lyon, Université de Lyon, UMR Inserm 1052 CNRS 5286, Centre Léon Bérard, 69008 Lyon, France; erw.eriau@gmail.com or; 2 Ecole Normale Supérieure de Lyon, 69342 Lyon, France; 3 Centre de Recherche des Cordeliers, Equipe 11 labellisée par la Ligue Nationale contre le Cancer, INSERM, Sorbonne Université, Université de Paris, 75006 Paris, France or juliette.paillet@crc.jussieu.fr (J.P.); kroemer@orange.fr (G.K.); 4 Gustave Roussy Cancer Campus, Metabolomics and Cell Biology Platforms, 94800 Villejuif, France; 5 Faculté de Médecine, Université Paris-Saclay, 91190 Kremlin-Bicêtre, France; 6 Institut Universitaire de France, 75005 Paris, France; 7 Pôle de Biologie, Hôpital Européen Georges Pompidou, Assistance Publique–Hôpitaux de Paris (AP-HP), 75015 Paris, France; 8 Suzhou Institute for Systems Medicine, Chinese Academy of Sciences, Suzhou 215163, China; 9 Department of Women’s and Children’s Health, Karolinska University Hospital, 17164 Stockholm, Sweden

**Keywords:** cancer immunotherapy, metabolism, fasting, caloric restriction, caloric restriction mimetics

## Abstract

**Simple Summary:**

Reduced food intake significantly enhances healthy lifespan in both model animals and humans, and decreases the incidence of cancer and other age-related diseases. This beneficial effect is mediated by the cellular knock-on effects of reduced food intake. Interestingly, these effects differ between cancer and healthy cells because cancer cells have peculiar metabolic requirements. Some compounds called “caloric restriction mimetics” are able to recapitulate the effects of reduced food intake without impacting the nutritional status. Reduced food intake and these mimicking agents are both able to enhance responses to some chemotherapies, as well as to some regimens combining chemotherapy and immunotherapy. There are encouraging preclinical data supporting the use of reduced food intake or caloric restriction mimetics as an adjuvant to cancer chemo-immunotherapies. Clinical data are sparse, but generally favorable, and additional trials are ongoing.

**Abstract:**

Caloric restriction and fasting have been known for a long time for their health- and life-span promoting effects, with coherent observations in multiple model organisms as well as epidemiological and clinical studies. This holds particularly true for cancer. The health-promoting effects of caloric restriction and fasting are mediated at least partly through their cellular effects—chiefly autophagy induction—rather than reduced calorie intake per se. Interestingly, caloric restriction has a differential impact on cancer and healthy cells, due to the atypical metabolic profile of malignant tumors. Caloric restriction mimetics are non-toxic compounds able to mimic the biochemical and physiological effects of caloric restriction including autophagy induction. Caloric restriction and its mimetics induce autophagy to improve the efficacy of some cancer treatments that induce immunogenic cell death (ICD), a type of cellular demise that eventually elicits adaptive antitumor immunity. Caloric restriction and its mimetics also enhance the therapeutic efficacy of chemo-immunotherapies combining ICD-inducing agents with immune checkpoint inhibitors targeting PD-1. Collectively, preclinical data encourage the application of caloric restriction and its mimetics as an adjuvant to immunotherapies. This recommendation is subject to confirmation in additional experimental settings and in clinical trials. In this work, we review the preclinical and clinical evidence in favor of such therapeutic interventions before listing ongoing clinical trials that will shed some light on this subject.

## 1. Introduction

Fasting and caloric restriction (CR) were shown in non-human primates to reduce the incidence of not only cancer but also metabolic diseases, arteriosclerosis, and neurodegeneration [1,2,3,4,5]—thus extending the healthspan. Furthermore, fasting and CR were shown in yeast, plants, worms, flies, and rodents to prolong lifespan [6,7,8] and reduce the incidence of a wide array of age-associated pathologies, notably malignant diseases [9]. Fasting-mimicking-diets (FMDs) reproduce the effects of fasting while maintaining a food supply, yet with a limited number of calories and a particular macronutrient composition, frequently poor in proteins, enriched in unsaturated fats, and with low to moderate proportions of carbohydrates. FMDs were shown in pilot trials to reduce risk factors associated with aging, diabetes, cardiovascular disease and cancer, without major adverse effects [10,11,12,13]. Of note, CR promotes both lifespan and healthspan, whereas exercise (and the associated increased calorie expenditure) has only been shown to prolong healthspan, [14] which hints at molecular effects of CR that go beyond its effects on energy balance. In humans, fasting is defined as a severe to complete deprivation of food intake (0–500 kcal/day) for several hours to days. We distinguish between continuous fasting, which can last over 40 consecutive days, and intermittent fasting, which exists under different patterns such as 16 h a day, 1 day out of 2, or 5 days per week [15,16]. By contrast, CR is typically defined as a long-term reduction by 10–50% of the recommended daily calorie intake [5,17]. FMDs are varied but usually involve at least short-term CR alongside other dietary modifications, often reduction in protein intake and sometimes supplementation with micronutrients such as vitamins. In this review, when indistinctively referring to fasting, CR, and/or their mimetics, we will use the term “energy reduction” (ER).

## 2. Clinical Evidence of the Health Benefits of ER

Inhabitants of the Okinawa island in Japan are renowned for their particularly long healthspan and lifespan, which is attributed by some to prolonged episodes of mild ER as well as consumption of nutrients exhibiting CR-mimicking properties [18]. The three-site CALERIE Phase 1 trial [19,20,21] was the first to investigate the effects of sustained (six months) CR in healthy volunteers as compared to various other metabolic interventions. On one site, patients undergoing prolonged CR showed a reduced pre-prandial insulin level and body temperature (two biomarkers of longevity in humans) [22]. CR had no impact on cortisol levels [23]. The CALERIE Phase 2 trial [24,25,26] followed 218 healthy non-obese volunteers across three institutions in a 2:1 randomized single-blind trial of a two-year 25% CR versus an ad-libitum control. CR significantly increased health-related quality-of-life (QOL) [27], decreased whole-body oxidative stress [28,29] (a marker of aging), and more generally slowed biological aging [30]. One of the smaller Phase 1 trials [20] showed that CR-induced weight loss was associated with a reduction in bone-density, an effect that was not associated with exercise-induced weight-loss [31]. However, this finding was not verified in the (larger) Phase 2 trial where no significant difference in adverse effects was witnessed between treatment and control groups [32]. This observation supports the safety of CR interventions. Moreover, prolonged moderate CR without exercise does not seem to compromise aerobic capacity [33]. Other trials have associated FMDs with improvements in a range of biomarkers of aging and age-related diseases [34].

Unsurprisingly, clinical trials revealed weight-loss proportionate to the intensity and duration of CR [11,22,35,36,37] and a corresponding increase in the percentage of fat-free mass [38]. Around half of the CR-induced weight-loss was maintained two years after completion of the intervention [39]. Moreover, baseline daily energy expenditure was reduced beyond the level expected from reduced metabolic mass, showing CR could induce metabolic adaptation [22,29,40]. This adaptation was shown to be accompanied by an increase in leptin levels [41] (although this did not change subjective ratings of appetite [42]). ER also improved metabolic flexibility (i.e., the capacity to adapt fuel oxidation to availability), which in turn improved sensitivity to insulin [43]. Furthermore, the CR group in the CALERIE trial showed significant improvements in metabolic disease risk factors (C-reactive protein [CRP], insulin sensitivity, and metabolic syndrome score) [44]. CR significantly decreased triiodothyronine levels as well [11], which is interesting as decreased thyroid function is associated with increased longevity in model organisms [45,46,47,48,49] and elderly humans [50,51,52]. In glucose-resistant overweight to obese patients, ER reduced insulin resistance and insulin-like growth factor 1 (IGF1) levels, [53] but this effect seems only transient [54] and limited to the weight-loss phase—except if accompanied by a reduction in protein intake [55]. This improves glucose tolerance, even though the intervention is modest [36]. Of note, no change in IGF1 (nor growth-hormone (GH)) levels was observed in non-obese volunteers [56]. Moreover, prolonged CR was shown to reduce liver enzymes (alkaline phosphatase and gamma-glutamyl transferase) and to increase levels of circulating bilirubin, all of which correlate with improved liver function [57].

Participants in the CALERIE trial [24] affected to the CR group showed a sustained reduction in all measures of cardiovascular risk (low-density lipoprotein [LDL]/high-density lipoprotein [HDL] cholesterol ratio, systolic and diastolic blood pressure) [44]. These effects were robust after controlling for weight loss-related changes, once again pointing to a molecular effect of ER beyond simple weight loss. Furthermore, prolonged CR was shown to markedly reduce risk factors for atherosclerosis (total cholesterol, LDL/HDL ratio, triglycerides, fasting glucose and insulin levels, CRP, platelet-derived growth factor AB), translating into a 40% reduction in carotid artery intima-media thickness [58]. CR was shown as well to improve the sympathetic/parasympathetic nervous system balance (a risk factor of cardiovascular diseases) in overweight individuals [59]. These effects translated to a 30% reduction of the 10-year risk of cardiovascular disease [54], confirming earlier projections [60]. In obese patients treated with at least one medication for hyperlipidemia, hypertension, or diabetes, total body fat and cardiometabolic risk factors were reduced by ER without increasing adverse events or lean mass loss [61].

In non-obese humans, CR reduced whole-body inflammation as measured by circulating markers such as lymphocyte counts, intercellular adhesion molecule-1 (ICAM-1, also known as CD54), serum CRP, tumor necrosis factor-alpha (TNF-α), [11] and leptin. However, CR had no effect on clinically significant indicators of cell-mediated immunity (such as delayed-type hypersensitivity skin response or antibody vaccine response) [62]. In infectious diseases, fasting-induced metabolism was shown to increase tissue tolerance to bacterial-induced inflammation, but not to viral-induced inflammation [63]. Moreover, prolonged cycles of fasting reverse immunosuppression caused by cytotoxicants [64]. Interestingly, spermidine, a polyamine whose administration mimics CR, was able to rescue the formation of the memory CD8^+^ T-cell compartment in aged mice [65]. Furthermore, an FMD [10] was shown to increase tumor immunogenicity and tumor-infiltrating lymphocytes, as well as to enhance the number of common lymphoid progenitor cells [66]. These observations are in coherence with previous results in long-lived non-human primates showing that ER reduced T-cell senescence [67]. These results should be considered in perspective with a recent study indicating that T-cell dysfunction in tumors is linked to the cellular distribution of acetyl-coenzyme A (AcCoA), [68] a criterion which is closely linked to ER, as we will see below. An ongoing clinical trial [69] should offer more information about the impact of FMDs on antitumor immunity.

A history of severe ER (due to anorexia nervosa) was shown to strongly reduce the incidence of breast cancer [70]. Such a protective effect of ER is known at least since 1914 [71]. It has been continuously investigated since [72], and there is a strong rationale supporting it [9,73,74,75,76,77,78]. As abovementioned, ER reduces aging [9,22,28,29,30], inflammation, [11,62], and metabolic imbalances [36,43,53], all of which are associated with cancer initiation and progression. Further, a higher intake of spermidine was shown in a prospective epidemiological study to be associated with lower general and cancer-specific mortality [79,80]. Despite this strong rationale and preclinical evidence, relatively few clinical investigations on ER in cancer treatment have been completed or are ongoing; most of them investigate FMD regimens [81]. Numerous clinical trials of dietary interventions are underway, with a focus on modifications to the nature of nutritional intake (e.g., ketogenic diet) [81].

There is clinical evidence that short-term fasting (STF) surrounding chemotherapy initiation is well tolerated [82,83,84,85] and is even reported to improve QOL and chemotherapy-associated fatigue [86]. Furthermore, STF appears to either reduce DNA damage or promote DNA reparation [83,84,87]. A recently published clinical trial [88] showed that stage II/III HER2-negative breast cancer patients benefited from an FMD consisting of a low amino acid short-term ER diet. Specifically, both radiological and pathological responses were statistically more frequent in patients using such an FMD [87]. However, this trial failed to meet its primary endpoint, which was attributed to low patient compliance with the FMD [89]. Most published clinical evidence in the field is from small-sized pilot studies, calling for upscaled evaluations of the effects of STF and CR in combination with standard-of-care. Such trials are underway [81,90,91,92].

**Figure 1:**
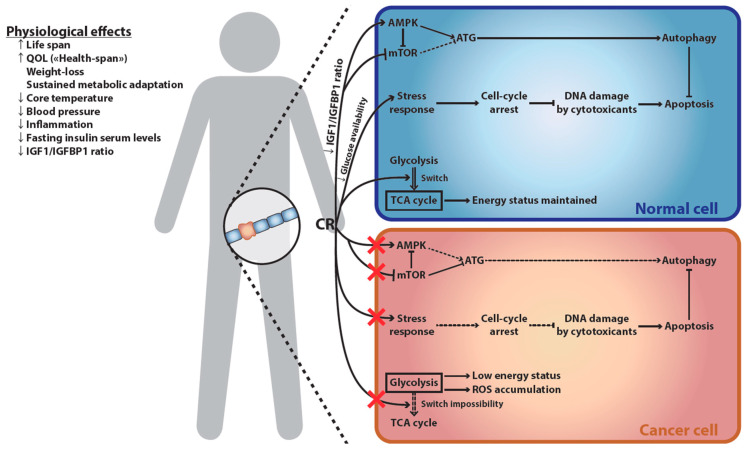
Cellular and physiological effects of ER. Consequences of ER at the physiological level (**left**) and its differential impact on normal versus cancer cells (**right**). ER has broad-ranging physiological effects, increasing both lifespan and QOL. This increase in the length and quality of life is strongly associated with ER-induced weight loss and sustained metabolic adaptation. The latter results in decreased markers of accelerated senescence, including core-body temperature, blood pressure, inflammation, fasting insulin serum levels, and IGF1/IGFBP1 ratio. This last parameter explains part of ER-mediated differential effect on cancer versus healthy cells: decreased IGF1/IGFBP1 ratio up-regulates AMPK, an activator of autophagy, and down-regulates mTOR, an autophagy inhibitor. Autophagy then protects the normal cell against chemotherapy-induced cell death. This cellular switch is not observed in cancer cells, which often have developed independence from the IGF1/IGFBP1 signal. Furthermore, ER reduces circulating glucose levels, which induces a cell-cycle arrest, thus protecting cells against chemotherapy-induced DNA damage. Cancer cells are often independent of cell-cycle arrest signals, and thus suffer increased DNA damages upon chemotherapeutic treatment. Finally, reduced glucose availability induces a reliance of normal cells on the TCA cycle for maintaining their energy status. This is not possible in most cancer cells, which rely on glycolysis to fuel their growth (“Warburg effect”) and are thus deprived of energy. As increased oxygen consumption fails to produce ATP, this leads to the accumulation of toxic ROS in cancer cells, an effect which has been referred to as the “anti-Warburg effect”. AMPK, AMP-activated protein kinase; ATG, autophagy-related proteins; ER, energy restriction; IGF, insulin-like growth factor; IGFBP, IGF-binding protein; mTOR, mammalian target of rapamycin; QOL, quality of life; ROS, reactive oxygen species; TCA, tricarboxylic acid.

Despite their multiple health- and life-span prolonging effects as well as their therapeutic potential in numerous diseases including cancer, patients—and people in general—have difficulties in adhering to calorie-restricting diets [36,93,94]. Furthermore, some of the health-promoting effects of ER may be mediated by its cellular consequences rather than weight reduction per se (whole body and cellular effects of ER are recapitulated in Figure 1). This led to the identification of caloric restriction mimetics (CRMs), a class of compounds that recapitulate some biochemical and physiological effects of fasting and CR [95]—in particular autophagy induction [96].

## 3. Differential Impact of ER on Healthy versus Cancer Cells

It has been known for more than a decade that starvation protects normal but not transformed cells against chemotherapeutics and oxidative damage, in yeasts, cell cultures, and mice [97,98]. This effect has been observed in several malignancies [99] such as colon carcinomas [100], melanomas [101], gliomas [101,102], and breast cancers [66,103]. Such phenomenon has been dubbed “differential stress resistance” (DSR; sometimes referred to as “differential stress sensitization” [99]). Proposed molecular mechanisms for DSR are recapitulated in Figure 1. DSR likely originates from the independence of malignant cells from growth signals and their insensitivity to anti-growth signals [97,104]; two hallmarks of cancer described by Hanahan and Weinberg [105,106]. These characteristics result from oncogenic gain-of-function mutations affecting the activity of AKT, mechanistic target of rapamycin (mTOR), RAS, and other pro-proliferative signaling factors, and/or of loss-of-function mutations in genes encoding tumor suppressors such as TP53. Consequently, cancer cells are unable to adapt to the lack of nutrients and maintain a sustained proliferation. On the contrary, normal cells switch to a maintenance program conferring resistance to stress. Fasting for 72 h protected melanoma and glioma-bearing mice against the toxicity associated with high-dose chemotherapy. This beneficial effect was mediated through a 70% decrease of the level of circulating IGF1, and a concomitant 11-fold increase of the plasmatic level of its inhibitor, insulin-like growth factor-binding protein 1 (IGFBP-1) [101]. Furthermore, ER reduces available glucose, which may affect cancer cells relying on glycolysis [107] (i.e., the “Warburg effect”) more than quiescent normal cells and reduces glycosylation of proteins [108]. On top of glucose restriction which bears more heavily on glycolysis-dependent cancer cells, STF was shown to increase oxygen consumption in cancer cells while failing to produce ATP through the respiratory chain. This phenomenon, termed the “anti-Warburg effect”, leads to the accumulation of reactive oxygen species, cellular damage, and sensitization of cancer cells to apoptosis upon exposure to chemotherapeutic agents [109]. Reduced protein glycosylation increases protein misfolding, which is handled through the unfolded protein response (UPR). As UPR is already upregulated in most cancer cells but not normal cells [110], this may prove an additional differential stressor of cancer cells. Finally, Valter Longo’s team has shown that heme oxygenase-1 (HO-1) acts as a critical mediator of the DSR. Upon FMD/STF, HO-1 expression increased in normal tissues but decreased in breast tumors. This reduction of HO-1 sensitized malignant cells in vitro and mammary murine tumors in vivo to chemotherapy. Accordingly, the overexpression of HO-1 appeared sufficient to impair treatment efficacy [66].

One of the knock-on effects of ER is autophagy induction, which is triggered in response to cellular stress such as DNA damage, ER or mitochondrial stress, oxidative or metabolic stress [111]. In cancer cells, autophagic activity helps to survive in the hypoxic and nutrient-deprived tumor microenvironment and has also been described as a drug resistance mechanism. Somewhat counter-intuitively, this knock-on autophagy induction is not detrimental to the general antitumoral effect of ER and helps explain DSR. As we have seen, cancer cells are more sensitive to metabolic stresses and, as we know, they are also more sensitive to genotoxic stress than most somatic cells. Thus, the concomitant amplification of these two stresses thanks to ER and chemotherapy can prove fatal to malignant cells, whereas ER-induced autophagy probably contributes to its observed protective effect against chemotherapy in healthy cells. Moreover, and perhaps most importantly, cellular stress and autophagy contribute to tumor immunogenicity. Therefore, their co-stimulation by cytotoxic drugs plus ER further promotes the invasion of the neoplastic microenvironment by dendritic cells [112], and in turn the initiation or reinstatement of cancer immunosurveillance [113,114,115].

## 4. Molecular and Cellular Signature of ER

Autophagy was first described in 1957 by Clarck [116], with the term being coined in 1963 by de Duve [117], and the associated machinery being elucidated in 1993 [118], for which Pr. Ohsumi was awarded the 2016 Nobel Prize in Physiology and Medicine [119]. Several types of autophagy have been identified [120], with the main one being macroautophagy (which will be referred to as “autophagy” for the scope of this review). Autophagy is a conserved mechanism that can both maintain cellular homeostasis by recycling dysfunctional organelles or proteins (including aggregates) from the cytoplasm [121] and regulate the cell’s energy stocks by converting macromolecules into energy-rich substrates [122]. Autophagy is also involved in redistributing key macronutrients but also ions such as iron [123], zinc [124], or potassium [125]. The interest of autophagy induction in the treatment of cancers is a hotly debated subject [126], with the consensus tending towards a benefit [127], particularly in light of its relationship to immunogenic cell death [128] (ICD), a concept which is discussed below.

Protein acetylation usually inhibits autophagy whereas protein deacetylation promotes autophagy. Mechanistically, in nutrient-rich conditions, mTORC1 (mTOR complex 1) phosphorylates and thus activates the protein acetyl transferase p300. In turn, phosphorylated p300 acetylates autophagy proteins ATG5 and ATG7, thus inhibiting autophagosome formation. Furthermore, p300 acetylates nuclear LC3 (microtubule-associated protein 1A/1B-light chain 3; known as ATG8 in yeasts), which impairs its interaction with DOR (diabetes and obesity-regulated gene) required for its export to the cytoplasm, and thus prevents its cytoplasmic interaction with ATG7 to promote autophagy. Conversely, starvation conditions stimulate deacetylation of ATG5, ATG7, and LC3 by sirtuins (SIRTs) [129,130]. The starvation of the amino acid leucine induces autophagy by a reduction of acetyl coenzyme A, thus inhibiting the p300-mediated acetylation of raptor, a component of mTORC1, and resulting in mTORC1 inhibition [131,132]. This points to intimate crosstalk between the two nutrient sensors EP300 and mTORC1.

Protein acetylation is regulated by acetyltransferases and deacetylases, as well as the availability of intracellular AcCoA. The so-called “master regulator” mTOR is implicated in numerous cellular processes and diseases, notably cancer [133]. mTOR is a key repressor of autophagy [134] both directly and by activating lysine acetyltransferases (KATs). mTOR is under the control of insulin and IGF1 through the phosphoinositide 3-kinase (PI3K)/protein kinase B (PKB; often referred to as AKT) pathway [135]. As we have seen, ER reduces IGF1 levels [53] (albeit transiently)—thus de-repressing autophagy. Furthermore, ER activates AMP-activated protein kinase (AMPK), a key nutrient sensor whose activity varies with the AMP/ATP ratio [136] (ADP may play a role as well [137]) and thus with nutritional status. Under low nutrient availability, AMPK activation promotes the activation of Ulk1, [138,139,140] a kinase regulating autophagic activity. Meanwhile, AMPK also inhibits mTOR [141] (and its downstream effectors [142]). Inversely, active mTOR inhibits AMPK–Ulk1 interaction [140]. Furthermore, SIRTs are NAD-dependent lysine deacetylases (KDACs). Under CR, mitochondrial respiration is upregulated and produces more NAD which in turn increases SIRT deacetylase activity [143,144]. It is further hypothesized that in mammals, ER may prevent the aging-associated accumulation of biotin, a strong inhibitor of SIRTs [144]. Finally, by reducing nutrients and energy, ER depletes cytosolic AcCoA, thus reducing its availability as a donor of acetyl groups and displacing the equilibrium of protein acetylation.

## 5. Identification of CRMs

Based on the aforementioned biochemical signature of ER, compounds stimulating autophagy through cellular protein deacetylation have been identified and defined as CR mimetics, CRMs (Table 1) [95,96,145,146]. Further, the definition of CRMs implies that they must not have significant off-target or knock-on effects on other pathways unrelated to ER.

Three main classes of CRMs can be distinguished depending on their ability to (1) inhibit acetyltransferases (e.g., EP300), (2) activate deacetylases (e.g., SIRT-1 or SIRT-3), or (3) deplete the cytosolic stock of AcCoA (e.g., through inhibition of the ATP citrate lyase (ACLY)). Multiple natural autophagy inducers can modulate the equilibrium of protein acetylation (Table 1) [95,145]. For instance, hydroxycitrate (extracted from the plants *Garcinia* or *Hibiscus*) and radicicol (produced by the fungus *Pochonia chlamydosporia*) are well-characterized inhibitors of ACLY [96,147,148,149,150,151,152,153,154,155]. Garcinol (also found in *Garcinia*), curcumin (extracted from curcuma), spermidine (a polyamine produced by cells to support proliferation and differentiation, and enriched in some vegetables, cereals, or soy derivatives [156]), as well as anacardic acid (extracted from the shell of the cashew nut) are inhibitors of EP300 [80,157,158]. Besides anacardic acid, other salicylate derivatives like aspirin reduce EP300 activity [159,160,161]. Moreover, monophenols like caffeic [162,163,164,165] and gallic [162,166,167,168,169] acids (common in plants) stimulate SIRT activity. The same property has been observed for several subtypes of polyphenols such as the stilbenes resveratrol and piceatannol (found in red wine or tea), or the flavonoids catechin, epicatechin, myricetin, and quercetin (enriched in tea, fruits). Similarly, nicotinamide (also known as vitamin B3/PP) and its derivatives (e.g., nicotinamide mononucleotide (NMN), nicotinamide adenine dinucleotide (NAD)) are SIRT activators [170,171]. Additional molecules with the propensity to mimic ER have been chemically synthesized. They include the specific inhibitors of ACLY SB-204990 [172,173], the activators of SIRT1 SRT1720 [174,175,176] and SRT2104 [177], as well as the inhibitors of EP300 A-485 [178] and c646 [179,180]. Other CRMs decrease the production of AcCoA by reducing the mitochondrial influx of metabolites fueling its synthesis. For instance, perhexilin inhibits carnitine O-palmitoyl transferase 1 (CPT1), thus altering the import of fatty acids from the cytosol [147,181] The synthetic agent UK5099 [182] and CPI-613 [183,184,185] hinder the activity of the mitochondrial pyruvate carrier (MPC) and pyruvate dehydrogenase (PDH), thus impeding the mitochondrial supply in pyruvate. In addition, the non-cleavable citrate analog, 1,2,3-benzene tricarboxylate, binds to the citrate transport carrier (CTP), prevents the mitochondrial export of citrate, and in turn represses the synthesis of AcCoA [186,187]. Other compounds, like 3,4′-dimethoxychalcone, potentiate the autophagic flux and decrease the acetylation profile of nucleo-cytosolic proteins through metabolic mechanisms that remain to be determined [188,189],

## 6. ER as an Adjuvant to Cancer Immunotherapy

As abovementioned, ER may be useful in the treatment of cancer, especially in combination with ICD-inducing therapies. Furthermore, the impact of fasting, CR, and their mimetics on tumor growth is at least partly immune-mediated. This begs the question of their application as an adjuvant to cancer immunotherapies. We recapitulate existing preclinical evidence of such combinations in Table 2. When screening the literature, we have to be mindful of publication bias (i.e., positive results are more likely to be published than negative results). However, as congruent results are reported with several different immunotherapies and ER regimens, we believe the available evidence generally confirms the interest of ER as an adjuvant to most types of cancer immunotherapies.

It is known since 1977 that some chemotherapies can stimulate the cytotoxic activity of immune cells in vitro, [244] which was confirmed in cancer patients in 1986 [245]. Nevertheless, chemotherapy-induced cell death (typically apoptosis) was largely considered tolerogenic or immunosuppressive, or at the very least null from the immune standpoint [246]. The concept of immunogenic cell death (ICD) emerged in the mid-2000s [247], challenging this view. In particular, our group demonstrated how tumor-specific immune responses determined the efficacy of some conventional cytotoxic chemotherapies [248], one reason for considering such cytotoxic agents as immunotherapies. Since this initial discovery, ICD has been widely studied and its mechanisms ascertained [249]. Broadly, pre-mortem endoplasmic reticulum stress [250,251] and autophagic activity [112] induce exposure of calreticulin [252,253] (CALR) on the surface of the plasma membrane during the pre-apoptotic stage, and ATP secretion [112] as well as the release of the nuclear protein high-mobility group box 1 (HMGB1) and the cytosolic protein annexin1 (ANXA1) during secondary necrosis [254]. In turn, ATP helps to recruit dendritic cells (DC) to the tumor bed [255]. ANXA1 binds formyl peptide receptor 1 (FPR1) thus facilitating DC homing to dying cells in the tumor bed, [256] and CALR stimulates tumor antigen engulfment by DCs [257]. HMGB1 then promotes activation of DCs [258]. All in all, this results in a strong interferon-γ-mediated immune response mainly involving cytotoxic CD8^+^ T-cells [259]—that is, immunosurveillance is reinstated. Furthermore, ICD induces a type-1 interferon response (probably through PRR sensing of the nucleic acids released from dying cells), which contributes to T-cell priming [260]. ICD status of chemotherapeutics is given growing attention in clinical practice, especially when it relates to combinations with immune checkpoints inhibitors (ICIs) [261].

Numerous cancer therapies currently used or under development are ICD inducers [262]. For instance, radiotherapy [263] is inducing ICD in an autophagy-dependent manner, [264] as are several chemotherapies: “classical” cytotoxic agents such as doxorubicin, [265] cyclophosphamide, [266,267] or oxaliplatin; [268,269] but also “targeted” therapies like crizotinib [270]. Interestingly, some combinations of non-ICD-inducing cytotoxic agents can result in the promotion of ICD, as is the case with carboplatin + pemetrexed bitherapy [271]. As a side note, cardiac glycosides exert an anticancer effect through ICD induction [272]. Interestingly, ICD can also be induced in cancer cells by physical means such as high hydrostatic pressure [273] or photodynamic therapy [253,274]. Other therapeutic approaches like oncolytic viruses [275] can precipitate ICD. Such effect was demonstrated with both RNA viruses such as the coxsackievirus B3 [276] or measles virus [277], and DNA viruses such as oncolytic adenoviruses [278,279] or herpes simplex viruses (HSV) [280]. Association of the latter with chemotherapeutic intervention even potentiated the immunogenicity of cancer cell death [281]. Additionally, an oncolytic peptide, namely LTX-315—displayed ICD-inducing properties as shown in vitro [282] and corroborated by preliminary clinical observations [283,284].

As we have seen, ICD induction is mostly autophagy-dependent, with autophagy contributing to ATP secretion and thus to DC recruitment, [112] a critical step for mediating an adaptive antitumor immune response. This has led several authors to propose the use of ER to further stimulate autophagy upon treatment with ICD-inducing therapeutic regimens; a hypothesis since validated by multiple preclinical investigations. For example, fasting synergizes in vitro with tyrosine kinase inhibitors (TKIs) like crizotinib [240,241] (which induces ICD [270]) and others [240] for the inhibition of tumor growth. Furthermore, fasting enhances the response of both radio- and chemo-therapy (temozolomide) on glioma models in mice [102]. Of note, standard clinical practice is to administer temozolomide in a fasted state. Our team has shown [113] that this synergistic effect of fasting on chemotherapies could be recapitulated by CRMs. The benefit of these ER interventions resulted from an enhanced infiltration of the tumor bed by activated CD8^+^ T cells, together with a concomitant reduction of regulatory CD4^+^ T lymphocytes from the tumor bed [113]. Further work has confirmed a critical involvement of CD11b^+^ myeloid cells and in particular an ER-induced enrichment in mature monocyte-derived DCs [236].

As treatment with cytotoxic agents increased expression of immunosuppressive programmed cell-death 1 (PD-1) and its ligand (PD-L1) on cancer or immune cells, we evaluated a triple combination of a CRM + an ICD-inducing chemotherapy + an anti-PD1 antibody. This tritherapy led to the complete cure of most tumor-bearing mice [236]. Combination regimens of ICIs with ER ought to be explored further. In this line, an interesting article [237] has recently reported that CRM resveratrol promoted abnormal glycosylation of PD-L1 and thus its endoplasmic retention. Consequently, cancer cells exposed to resveratrol demonstrated enhanced sensitivity to T cell-mediated killing. Another recent study [285] showed deacetylation-dependent translocation of PD-L1 from the cell membrane to the nucleus. There, PD-L1 bound to DNA and promoted the expression of genes related to antigen presentation and inflammation, but also of other immune checkpoints such as V-domain immunoglobulin suppressor of T cell activation (VISTA) and PD-L2. Although the exact consequences of membrane PD-L1 deacetylation remain unclear [286], these results provide further evidence of a likely positive interaction between ER and ICB.

Generally, the combination of fasting, CR, or their mimetics with immunotherapies has been somewhat understudied so far, given the strong rationale supporting this approach. For example, treatment with interleukin-2 (IL-2) is a long-existing immunotherapeutic approach to melanoma and metastatic renal cell carcinoma [287]. ER was shown by one team in the 1980′s as promoting thymocyte proliferation in response to exogenous IL-2 in autoimmune-prone mice [239]. Yet, associations of ER and IL-2 do not seem to have been studied since.

Oncolytic virotherapy is considered a promising strategy for cancer treatment, [288] as it causes both targeted cytotoxicity and anticancer immune responses. Regrettably, few publications have investigated combinations of ER with oncolytic virotherapy. However, recent evidence [243] has emerged that transient fasting increased the replication and glioblastoma-specific cytotoxicity of herpes simplex virus (HSV), which is being engineered as an oncolytic virus, but did not stimulate HSV-induced killing of normal astrocytes. This differential effect was convincingly explained by the authors by depressed protein synthesis upon fasting (mediated by increased phosphorylation of the translation initiation factor eIF2α) in normal cells but not cancer cells. Even more recent evidence [242] has emerged in favor of a similar positive effect of fasting on the efficacy of an oncolytic measles vaccine on colorectal cancer cells. However, the effect of autophagy on replication and immunogenicity seems to differ, depending on the virus [289,290]. It is tempting to speculate that current nutrient-rich cell culture practices do not reflect the specific metabolic profile of the tumoral microenvironment. Thus, high-glucose, high glutamine media may skew in vitro studies of oncolytic viruses by providing metabolic conditions which favor (or hinder) viral replication/cytotoxicity but that are not available in the tumor microenvironment [291].

Some active fields of immunotherapeutic research have not yet been evaluated with respect to possible combinations with CR. Cancer vaccines have recently garnered renewed interest [292]. Unfortunately, there is no reported evidence of a combination of ER with any established cancer vaccine, despite evidence that ER promotes memory T cell function [293] and that the efficacy of a vaccine based on a sarcoma cell lysate could be increased in an autophagy-dependent fashion [294]. Adoptive cell transfer is another active field of cancer research, [295] with recent clinical successes. Yet, no combination with ER has been reported, despite recent reports [68] that tumor-infiltrating lymphocytes enter an autophagy-induced adaptive stem-like state which helps in maintaining their anti-tumor potential. This effect could in particular be manipulated (notably by CR) to maintain replicative potential in the case of autologous cell transfer [296]. Finally, there are no reports of combinations of ER with oncolytic peptides.

## 7. Ongoing/Reported Clinical Investigations on the Topic

Ongoing and reported clinical investigations combining ER with immunotherapeutic approaches are reported in Table 3. In most ongoing trials, an ICD-inducing chemotherapeutic agent is induced. The ER arm of the trial mostly consists of an FMD, which involves reduced caloric intake with a qualitative change in diet, usually an increase in the proportion of fats (i.e., a “ketogenic diet”) or a restrictive feeding schedule (i.e., “intermittent fasting”). We believe these regimens to be sub-optimal for cancer patients as the reduced energy intake can be difficult to sustain, both psychologically and physiologically. By contrast, CRMs recapitulate ER biochemical effects while allowing for ad libitum feeding. Therefore, CRMs offer two advantages: (i) they do not compromise the nutritional status of cancer patients, who are often at risk of cachexia, and (ii) they are psychologically easier to tolerate and hence ensure better treatment adherence. At last, ER is being explored in two ongoing trials as a single agent, which will allow the evaluation of its specific (i.e., not as part of a combination) contribution to clinical outcomes (Table 3).

## 8. Conclusions

ER has a differential effect on cancer and healthy cells, a process named “differential stress resistance”. This effect is mediated in part by the different metabolic requirements of cancer and healthy cells, but also—and perhaps chiefly—by the cellular effects of ER, especially as it pertains to autophagy.

We have known for a few years that autophagy promotes (i) cancer cell immunogenicity, (ii) tumor-bed immune infiltration, and (iii) depletion of tumor-infiltrating regulatory T cells, especially when autophagy is induced at the same time as ICD-inducing agents are administered. Interestingly, autophagy induction can be mimicked pharmacologically using CRMs. These mimetics recapitulate the anticancer immune effects of ER and synergize with a combinatorial treatment composed of ICD inducers and a PD-1 blockade to provoke complete tumor regression in a murine model of fibrosarcoma.

Recent work by Vodnala et al. [68] has shed further light on the relationship of CR with immunity in the context of cancer by showing how a calorie-restricted state promotes T-cell stemness and helps in maintaining cancer immunosurveillance. This is especially interesting considering older work by Puleston et al. [65] showing the central role of autophagy in the formation of memory T CD8^+^ cells.

These works and others all point towards an immune mechanism that explains the capacity of ER to prevent—and probably event to treat—cancer. This has led us and others to propose ER as an adjuvant to immunotherapy, especially as ER is relatively well tolerated. We can thus only regret the relative lack of published work on such combinations, despite globally positive results in reported experiments. It is our hope that these issues will be addressed by ongoing clinical trials as well as by preclinical research on combinations of ER with certain immunotherapeutic approaches, and especially ICIs, oncolytic viruses, and cancer vaccines.

## Figures and Tables

**Table 1:** Some non-toxic compounds inducing autophagy through protein deacetylation and referred to as CRMs.

CRM	Target(s)	Reference(s)
**1,2,3-benzene tricarboxylate**	CTP	Cappello et al. [186]; Guay et al. [187]
**3,4′-dimethoxychalcone**	GATA-1/2, TFEB, TFE3	Carmona-Guttierez et al. [189] Chen et al. [188]
**A-485**	EP300	Lasko et al. [178]
**Anacardic acid**	EP300	Rajendran et al. [190]; Wu et al. [191]; Pietrocola et al. [192]
**Aspirin**	AMPK, EP300, COX-1, COX-2, mTOR, NF-kB	Pietrocola, Castoldi, et al. [159,160,161]
**C646**	EP300	Mariño et al. [179]; Liu et al. [180]
**Caffeic acid; Caffeic acid ethanol amide; Caffeic acid phenethyl ester**	AMPK, Sirtuins (notably SIRT1 and SIRT3)	Pietrocola et al. [162]; Lee et al. [163]; Treviño-Saldaña & Garcia-Rivas [164]; Murtaza et al. [165]
**Catechin**	Unknown	Chung et al. [193]; Pietrocola et al. [162]
**CPI-613 (lipoate analog)**	PDH	Stuart et al. [184]; Zachar et al. [183]; Lee et al. [185]
**Cyclopentylidene-[4-(4-chlorophenyl)thiazol-2-yl)hydrazone (CPTH-2)**	HATs	Mai et al. [194]; Chimenti et al. [195]
**Curcumin**	AMPK, mTOR, EP300	Kubota et al. [196]; Shakibaei et al. [197]; Ryu et al. [198]; Suckow & Suckow [199]; Liao et al. [200]; Seo et al. [201]; Sharma et al. [202]; Rao et al. [203]; Huang et al. [204]; Bimonte et al. [205]; Balasubramanyam et al. [206]; Neckers et al. [207]; Morimoto et al. [208]; Sunagawa et al. [209]; Kang et al. [210]; Chung et al. [193]; Pietrocola et al. [192]
**Epigallocatechin-3-gallate (EGCG)**	AMPK, mTOR, HATs, KDACs	Chen et al. [211]; Jang et al. [212]; Niu et al. [213]
**Epicatechin**	Unknown	Chung et al. [193]; Pietrocola et al. [162]
**Gallic acid**	AMPK and HATs	Jara et al. [166]; Lu et al. [167]; Kim et al. [168]; Quideau et al. [169]; Pietrocola et al. [162]
**Garcinol**	EP300	Chen et al. [157]; Li et al. [214]; Arif et al. [215]; Pietrocola et al. [192]
**Hydroxycitric acid**	ACLY	Phan et al. [147]; Hanai et al. [148]; Asghar et al. [149]; Onakpoya et al. [150]; Mariño et al. [179]
**MB-3**	HATs	Mai et al. [194]
**Myricetin**	SIRT1	Pietrocola et al. [162]
**Nicotinamide and derivatives (NAD+; nicotinamide mononucleotide; nicotinamide riboside)**	Sirtuins	Lu et al. [170]; Belenky et al. [171]
**Perhexiline maleate**	CPT1, mTOR	Phan et al. [147]; Abozguia et al. [181]
**Piceatannol**	SIRT1	Yum et al. [216]; Kinoshita et al. [217]; Kwon et al. [218]; Minakawa et al. [219]; Pietrocola et al. [162]
**Quercetin**	SIRT1	Angst et al. [220]; Pratheeshkumar et al. [221]; Dong et al. [222]; Chung et al. [193]; Pietrocola et al. [162]
**Radicicol**	ACLY, HSP90	Zhao et al. [151]; He et al. [152]; Sonoda et al. [153]; Conte et al. [154]; Griffin et al. [155]
**Resveratrol (SRT501 = micronized resv.)**	SIRT1, AMPK, NF-κB	Timmers et al. [223]; Lissa et al. [224]; Minor et al. [174]; Pearson et al. [225]; Liu et al. [226]; Fiori et al. [227]; Hubbard et al. [228]; Park et al. [229]; Chung et al. [193]; Pietrocola et al. [162]; Milne et al. [175]
**SB-204990**	ACLY	Hatzivassiliou et al. [172]; Pearce et al. [173]
**Spermidine**	EP300, mTOR, AMPK	Eisenberg et al. [230]; Bauer et al. [231]; Soda et al. [232]; Matsumoto et al. [233]; Paul & Kang [234]; LaRocca et al. [158]; Pietrocola et al. [192]
**SRT1720**	SIRT1	Minor et al. [174]; Milne et al. [175]; Mitchell et al. [176]
**SRT2104**	SIRT1	Hoffmann et al. [177]
**Trientine (TETA)**	SAT1	Pietrocola et al. [235]
**UK5099**	MPC	Bricker et al. [182]

Abbreviations. ACLY, adenylate citrate lyase; AMPK, adenosine monophosphate-activated protein kinase; COX, cyclooxygenase; CPT1, carnitine O-palmitoyl transferase 1; CRMs, caloric restriction mimetics; CTP, mitochondrial citrate transport carrier; EP300, Histone acetyltransferase p300; GATA, transcription factor of the GATA-motif binding zinc-finger protein family; HAT, histone acetyltransferase; HSP, heat-shock protein; KDAC, lysine deacetylase; MPC, mitochondrial pyruvate carrier; mTOR, mammalian target of rapamycin; NAD, nicotinamide adenine dinucleotide; PDH, pyruvate dehydrogenase; SIRT, sirtuin; TFE3, transcription factor E3; TFEB, transcription factor EB.

**Table 2:** Reported regimens associating ER to immunotherapies in preclinical studies.

Type of Immunotherapy	Therapeutic Agent	Fasting	FMD	CR	CRM
**Immune checkpoint modulators**	Anti-PD-1	[236]	-	-	[236]
	Anti-PD-L1	-	-	-	[237]
	Anti-TNFRSF4	-	-	[238]	[238]
**Interleukin**	IL-2	-	-	[239]	-
**ICD-inducing chemotherapies**	Cyclophosphamide	[66,101]	[66]	-	-
	Doxorubicin	[66,101]	[66]	-	-
	Mitoxantrone	[113,236]	-	-	[113,236]
	Oxaliplatin	[109,113,236]	-	-	[113,236]
	Tyrosine kinase inhibitors	[240,241]	-	-	-
	Temozolomide	[102]	-	-	-
**Oncolytic viruses**	Measles virus	[242]	-	-	-
	Herpes simplex virus	[243]	-	-	-

Cells indicate the references of the articles reporting the corresponding combinations together with the presence or absence of reported antitumor efficacy in green or red, respectively. CR, caloric restriction; ER, energy restriction; FMD, fasting-mimicking diet; ICD, immunogenic cell death; IL, interleukin. PD-1, programmed cell death 1; PD-L1, programmed cell death ligand 1; TNFRSF4, tumor necrosis factor receptor superfamily, member 4.

**Table 3:** Ongoing clinical trials listed on clinicaltrials.gov investigating ER-mediated metabolic reprogramming alone or as an adjuvant to immunotherapy, ensured by ICD inducers and/or ICIs.

Type of Combination Treatment	Cancer Type	ICD Inducer	ICI	ER	Reference
**ER**	Breast; Melanoma	-	-	FMD (5 days), for 1 or 4 four-weeks cycle(s)	NCT03454282
	Glioma	-	-	Fasting (72 h)	NCT04461938
**ER + ICD** **inducer**	Glioblastoma	Radiotherapy [263]	-	CR with ketogenic diet (72 h), followed by fasting (72 h), ultimately followed by another CR with ketogenic diet (72 h)	NCT01754350; Voss et al. [297]
	Breast	Doxorubicin [265] + cyclophosphamide [266,267]	-	CR 3 days prior to, 1 day following, and during the 12 weeks of chemotherapy	NCT01802346; Garg et al. [298]
	Breast	Radiotherapy [263]	-	25% CR from W-2 to W + 2 of a 6 week radiation therapy regimen)	NCT01819233; [299]
	Breast	Doxorubicin [265] + cyclophosphamide [266,267]	-	Short-term FMD	NCT02126449; BOOG group [87]
	Lung	Cisplatin/carboplatin + pemetrexed [271]	-	Metformin (1500 mg/day) +/- FMD (5 days every 3 weeks for 4 cycles)	NCT03709147
	Colorectal	Unspecified; likely includes oxaliplatin [268,269]	-	FMD (24 h before and after chemotherapy)	NCT04247464
**ER + ICI**	Skin	-	PD(L)-1 inhibitors	Fasting (48 h before treatment and 24 h after), for up to 3 three-weeks cycles	NCT04387084
**ER + ICD** **inducer + ICI**	NSCLC	Carboplatin + pemetrexed [271]	Pembrolizumab	FMD (from D-3 to D + 1 for 4 cycles)	NCT03700437

ER, energy reduction; ICD, immunogenic cell death; ICI, immune checkpoint inhibitor.
